# Genetic Dissection of Adaptation Traits in Apricot Through GWAS and QTL Analyses

**DOI:** 10.3390/ijms27146264

**Published:** 2026-07-14

**Authors:** Juan Alfonso Salazar, Germán Ortuño-Hernández, Álvaro Delgado, Mónica Moya-Andreo, David Ruiz, Pedro Martínez-Gómez

**Affiliations:** Fruit Breeding Group, Department of Plant Breeding, CEBAS-CSIC (Centro de Edafología y Biología Aplicada del Segura-Consejo Superior de Investigaciones Científicas), Campus Universitario Espinardo, E-30100 Murcia, Spain; jasalazar@cebas.csic.es (J.A.S.); gortuno@cebas.csic.es (G.O.-H.); adelgado@cebas.csic.es (Á.D.); mmoya@cebas.csic.es (M.M.-A.); druiz@cebas.csic.es (D.R.)

**Keywords:** chilling requirements, dormancy, GWAS, phenology, *Prunus armeniaca*, SNP

## Abstract

Understanding the genetic basis of adaptation traits including chilling requirements, flowering and fruiting is essential for developing apricot cultivars adapted to changing climatic conditions and for extending the apricot production calendar. The objective of this study is to detect and finely identify marker−trait associations linked to these adaptation traits including chilling requirements in apricot, using an R-based workflow developed with agroclimatic functions. In this study, high-density GBS-based linkage maps previously developed for two biparental populations (‘Bergeron’ × ‘Currot’ and ‘Goldrich’ × ‘Currot’) were used to analyze the genetic basis of key adaptation traits, including chilling requirement (CR), blooming date (BD), fruit development period (FDP), and ripening time (RT), through Genome-Wide Association (GWAS) and Quantitative Trait Locus (QTL) analyses. Phenotypic evaluation over eight years revealed wide variability across genotypes and strong correlations between CR and BD, particularly when using Chill Portions as a metric. Genome-wide association and QTL mapping consistently identified major loci on linkage group (LG) 1 for BD and CR, and on LG4 for FDP and RT, explaining up to 59% of phenotypic variance. The candidate gene (*qMD4.1 ANAC072*), upon analysis, revealed the involvement of epigenetic regulators, cold-responsive proteins, and transcription factors, offering plausible functional links between genotype and phenotype. These findings provide novel insights into the genetic control of dormancy and phenological traits in apricot and represent a valuable genomic resource for marker-assisted breeding programs aimed at improving climatic resilience and extending the harvest.

## 1. Introduction

**Figure 1:**
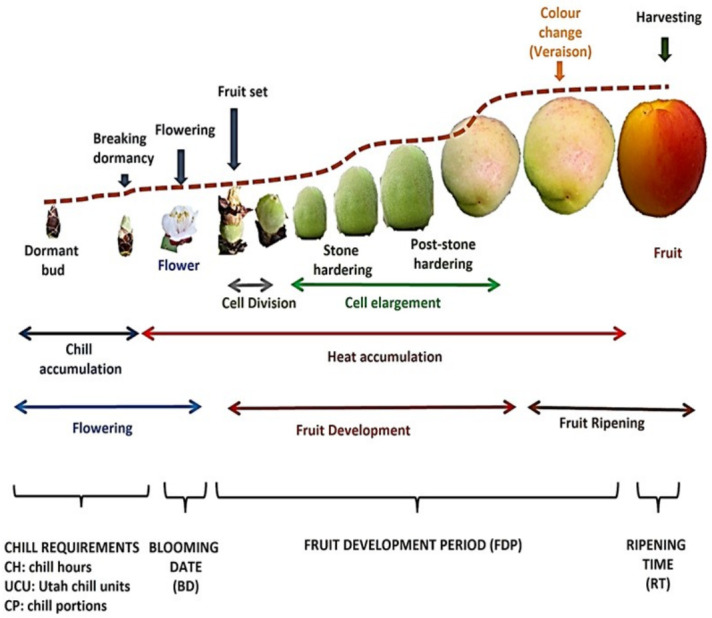
Schematic representation of adaptation traits in apricot flowering and fruiting.

Apricot (*Prunus armeniaca* L.) is a fruit species native to China, valued for its economic importance, which stems from its nutritional and industrial value, providing health benefits, along with its appealing aroma and flavor, which are appreciated by consumers. Widely cultivated in temperate regions worldwide, the species is noted for its high fruit quality and broad genetic variability [1]. Adaptation traits greatly influence flowering and fruit maturation periods and vary depending on geographic location and climatic conditions, which are mainly determined by chilling requirements (CRs) and the length of the fruit development period of each cultivar. In the Northern Hemisphere, such as in Spain or Turkey—two major apricot-producing countries—flowering typically occurs between March and April, with fruit maturing from May to July. Conversely, in Southern Hemisphere regions like Chile, flowering usually begins in September, and fruit is harvested from November to January [2] (Figure 1).

Deciduous fruit trees native to temperate regions undergo a period of dormancy during the winter, which enables them to withstand adverse climatic conditions [3]. Exiting this dormant state and achieving uniform and optimal flowering in spring requires each cultivar to accumulate a specific amount of CR, which is genetically determined. Therefore, the strategic selection of cultivars suited to local climates—where they can reliably satisfy their CR annually—has become essential for maintaining consistent yields and economic viability in fruit production systems [4].

Historically, the CR of *Rosaceae* species such as apricot, peach, sweet cherry, almond, apple, and pear has been determined using the traditional “forcing method”. This technique involves the weekly collection of shoots during the dormant period, followed by their exposure to standardized conditions in growth chambers for a predetermined duration [5]. Despite its accuracy, this method is labor-intensive, time-consuming, and limits large-scale genotype screening.

To overcome these limitations, statistical approaches that integrate long-term phenological records and temperature data have been proposed. In particular, Partial Least Squares (PLS) regression has emerged as a widely used method to identify the effective chilling accumulation period and to quantify genotype-specific chilling requirements with reasonable accuracy [6,7,8].

Temperate fruit tree breeding programs typically pursue two main objectives depending on the cultivation area. In mild-winter regions, the focus is on breeding and selecting low-chill (i.e., early-flowering) genotypes that can reliably meet their chilling requirements every year. In contrast, in colder regions, breeders aim to reduce the risk of frost damage during flowering by selecting high-chill (i.e., late-flowering) genotypes [9,10]. Accurate knowledge of these chilling needs can be used to determine the most suitable planting locations by correlating them with the specific agroclimatic conditions of a given region, particularly in the context of climate change, which is altering traditional patterns of chill accumulation and frost occurrence [11]. Additionally, extending the fruit harvest season—through the development of both early- and late-maturing cultivars—is essential for supporting market growth. Achieving these goals could be facilitated by the implementation of molecular markers, which are developed from the identification of highly significant quantitative trait loci (QTLs), as described in other fruit trees [12].

Genotyping-by-Sequencing (GBS) is a cost-effective, high-throughput technique that enables the identification of single-nucleotide polymorphisms (SNPs). Its high marker density has greatly enhanced the resolution of genetic linkage maps, making it a powerful tool for identifying QTLs and uncovering marker-trait associations in segregating populations—applications that are critical for plant breeding programs [13]. Within the *Prunus* genus, GBS has been widely applied in species such as *Prunus armeniaca* (apricot) [14], *P. domestica* (European plum) [15], *P. persica* (peach) [16], *P. avium* (sweet cherry) [17], and *P. dulcis* (almond) [18].

Advances in linkage mapping and QTL analysis have considerably enhanced the understanding of trait inheritance, thereby facilitating the discovery of genes associated with agronomically relevant characteristics. In particular, flowering time in apricot and peach has been demonstrated to follow a quantitative pattern of inheritance [19]. Using an apricot cross progeny, a QTL for flowering time was identified on linkage group (LG) 5 and was consistently detected across three consecutive years of evaluation [20]. In a backcross population between peach and *Prunus davidiana*, two QTLs for blooming date were mapped on LG1 and LG2 over two years, whereas additional QTLs on LG5 and LG6 were observed only in a single year [21]. In peach, a flowering time QTL on LG4 was found to be stable across a five-year period [22]. Moreover, two major QTLs for blooming date were mapped on LG1 and LG7, and these were shown to co-localize with major QTLs associated with chilling requirement, indicating a possible genetic relationship between the two traits [23]. More recently, flowering and maturity dates were evaluated in five progenies derived from peach, apricot, and sweet cherry over periods ranging from three to eight years [24]. For flowering date, major QTLs were consistently detected on LG4 in apricot and sweet cherry and on LG6 in peach, while additional QTLs were mapped on LG2, LG3, LG4, and LG7 across the three species. With respect to ripening date, a major QTL was consistently identified on LG4 in all three species, highlighting its central role in the genetic control of this trait.

Considering the lack of molecular markers linked to adaptation traits in apricot, the objective of this study is to detect and finely identify marker–trait associations linked to key phenological traits including chilling requirements in apricot, using an R-based workflow developed with agroclimatic functions. On the other hand, high-density genetic linkage maps developed from two biparental populations, ‘Bergeron’ × ‘Currot’ (‘B × C’) and ‘Goldrich’ × ‘Currot’ (‘G × C’), were used. Additionally, candidate genes located within these highly significant genomic regions will be identified. The outcomes of this research are expected to support the development of molecular markers for use in apricot breeding programs, thereby enabling marker-assisted selection and promoting the faster development of cultivars well-adapted to current agronomic and climatic challenges.

## 2. Results

### 2.1. Phenotypic Data Analysis

The analysis of phenotypic distributions showed broad variability for BD, RT, and FDP across genotypes in both ‘B × C’ and ‘G × C’ populations (Figure S1). In ‘B × C’, BD ranged from 49 to 93 Julian days, RT from 146 to 188, and FDP from 66 to 116. Similarly, in ‘G × C’, BD spanned from 44 to 88, RT from 130 to 180, and FDP from 57 to 113. On the other hand, CP (chill portions) values ranged approximately from 37 to 84 in both populations. When separating the two periods (2012–2014 vs. 2021–2025), these values range from 45 to 84 (2012–2014) and from 37 to 73 (2021–2025), indicating lower chill portions in the most recent years. These ranges reflect the wide phenological diversity present within the segregating populations, with the ‘G × C’ population clearly exhibiting earlier ripening times. To explore more in depth the relationship between chilling requirements and flowering time, Pearson correlation analyses were performed between BD (BD12 to BD25) and three chilling models: CH (chill hours), UCU (Utah chill units), and CP (Figure S2).

In both populations, a clear pattern emerged: later blooming dates (e.g., BD25) were positively correlated with higher chilling accumulation, particularly under the Chill Portions model, which showed correlation coefficients as high as 0.87 in ‘B × C’ and 0.80 in ‘G × C’. This suggests that genotypes requiring more chilling tend to bloom later in the season. In contrast, the opposite effect showed weak or low-magnitude negative correlations.

### 2.2. Marker-Trait Association Analysis (GWAS)

The SNP dataset obtained from previous apricot GBS studies was used to establish marker–phenology associations with the phenotypic dataset in the apricot populations, focusing on the most prominent associations for each trait and population.

**Figure 2:**
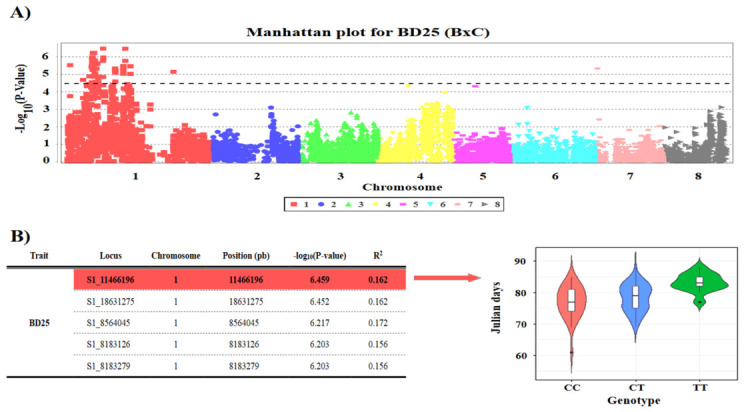
(**A**) Manhattan plot of genome-wide association analysis for BD in ‘B × C’ population. (**B**) Table of the most significant SNPs and trait distribution by SNP genotype in violin plot.

In the ‘B × C’ population, the Manhattan plot for BD25 revealed a moderate but clear association signal on chromosome 1, with several SNPs surpassing the genome-wide significance threshold. The most significant marker, S1_11466196, showed a −log_10_(*p*-value) of 6.459 and an R^2^ of 0.162, indicating a moderate contribution to phenotypic variance. The violin plot for this SNP showed a progressive increase in flowering time (Julian days) from genotype CC to TT, supporting a genotypic effect on bloom timing (Figure 2).

**Figure 3:**
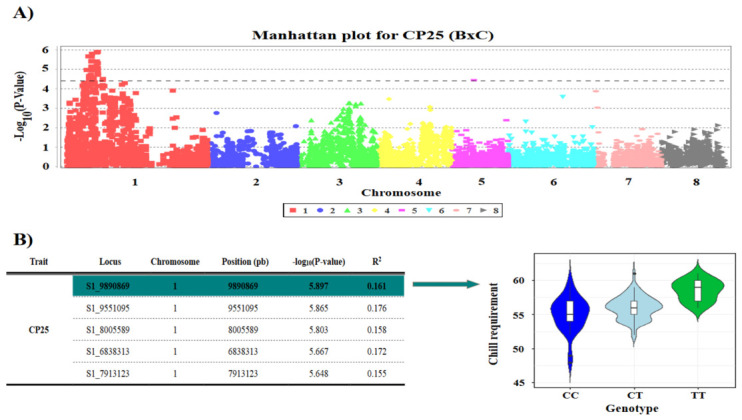
(**A**) Manhattan plot of genome-wide association analysis for CP in ‘B × C’ population. (**B**) Table of the most significant SNPs and trait distribution by SNP genotype in violin plot.

In terms of CP25 in the same population, the strongest association also mapped to chromosome 1, with SNP S1_989069 reaching a −log_10_(*p*-value) of 5.897 and R^2^ = 0.161. Several nearby SNPs displayed similar significance levels. The violin plot showed increasing chilling requirements from CC to TT genotypes, suggesting additive effects of alleles on chill accumulation level (Figure 3).

**Figure 4:**
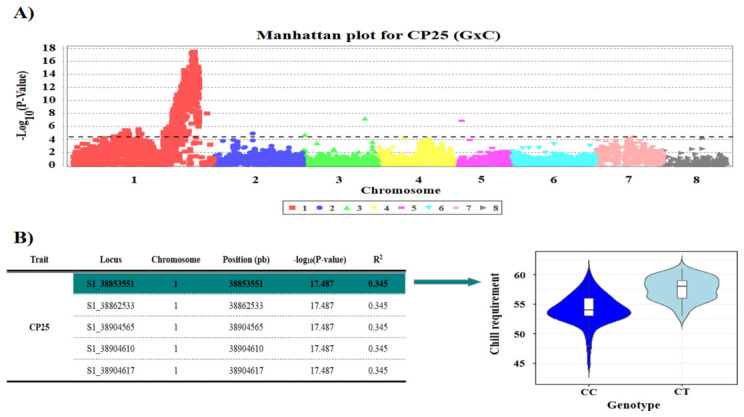
(**A**) Manhattan plot of genome-wide association analysis for CP in ‘G × C’ population. (**B**) Table of the most significant SNPs and trait distribution by SNP genotype in violin plot.

As for ‘G × C’ population, the marker–trait association for CP25 detected a highly significant region on chromosome 1, with a sharp peak centered at S1_38853551, showing a −log_10_(*p*-value) of 17.487 and an R^2^ of 0.345. This region was also supported by several closely linked SNPs showing identical −log_10_(*p*-value) and R^2^ values, reflecting their strong linkage disequilibrium within the same genomic region and, consequently, the same association signal. According to the violin plot, the CT genotype displayed a higher median chilling requirement than CC, highlighting a clear genotypic differentiation (Figure 4).

**Figure 5:**
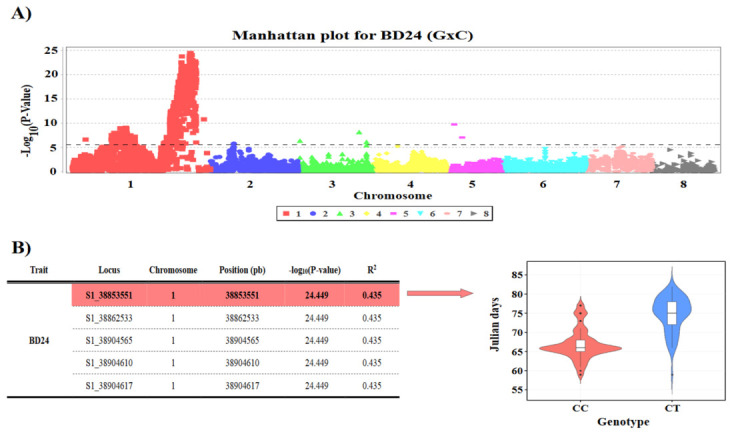
(**A**) Manhattan plot of genome-wide association analysis for BD in ‘G × C’ population. (**B**) Table of the most significant SNPs and trait distribution by SNP genotype in violin plot.

Regarding BD24, a strong and sharp association was also identified on chromosome 1, overlapping with the region associated with CP25. The most significant marker, S1_38853551, showed a −log_10_(*p*-value) of 24.449 and explained 43.5% of the phenotypic variance (R^2^ = 0.435). The violin plot illustrated that the CT genotype was associated with a markedly later blooming date compared to CC, confirming the major effect of this locus (Figure 5).

**Figure 6:**
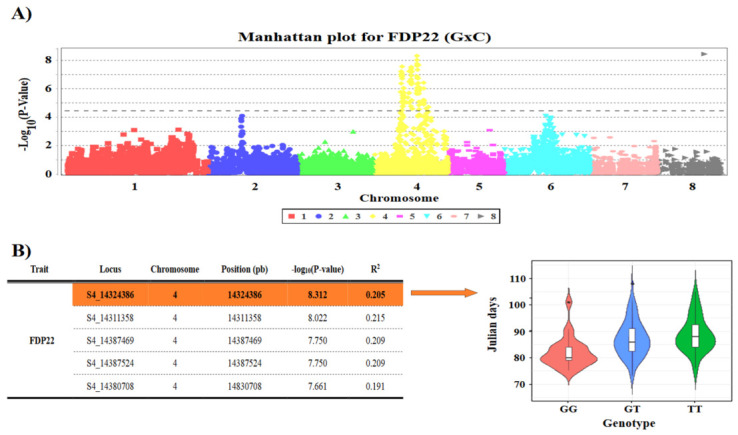
(**A**) Manhattan plot of genome-wide association analysis for FDP in ‘G × C’ population. (**B**) Table of the most significant SNPs and trait distribution by SNP genotype in violin plot.

Additionally, significant associations were found for FDP22 on chromosome 4, with the leading SNP, S4_14324386, showing a −log_10_(*p*-value) of 8.312 and an R^2^ of 0.205. Other markers within the same region showed similar signals. The violin plot revealed a trend where TT genotypes were associated with longer fruit development periods, suggesting a genotype-dependent extension of the FDP trait (Figure 6).

**Figure 7:**
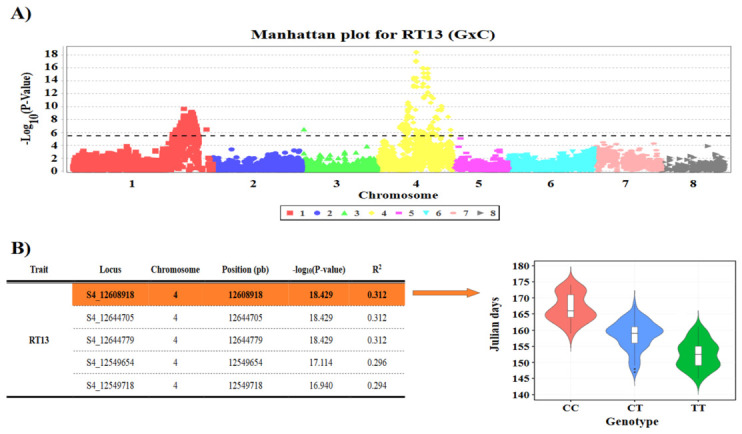
(**A**) Manhattan plot of genome-wide association analysis for RT in ‘G × C’ population. (**B**) Table of the most significant SNPs and trait distribution by SNP genotype in violin plot.

Finally, in the analysis of RT13, a well-defined peak was observed on chromosome 4, with S4_12608918 reaching a −log_10_(*p*-value) of 18.429 and R^2^ = 0.312. Several flanking SNPs supported the signal. The violin plot showed that TT genotypes matured earlier (lower Julian days) compared to CC, indicating a significant effect of this region on ripening time (Figure 7).

### 2.3. QTL Mapping

The QTL analysis revealed several significant loci associated with key phenological traits: BD, RT, FDP, and chilling requirements (i.e., CH, UCU and CP). The detection threshold was determined using permutation tests for each trait and year. The most consistent and relevant QTLs are summarized below, with their positions mapped across parental linkage maps for the ‘B × C’ (Figures S3 and S4) and ‘G × C’ populations (Figures S5 and S6).

#### 2.3.1. ’B × C’ Population

In the ‘Bergeron’ parent, multiple QTLs for BD were distributed across LG1, LG4, and LG7 (Table 1). A stable QTL on LG1 at 21.014 cM (marker S1_9890869) was consistently detected across three years (BD21, BD24, BD25), with LOD values up to 7.3 and explaining up to 23.1% of phenotypic variance, indicating a strong genetic control over flowering time.

For RT and FDP, a cluster of robust QTLs was identified on LG4 at ~48.8 cM, particularly at marker S4_12380084, significantly associated with both RT and FDP across multiple years. This locus reached LOD values of 9.4 for RT and 9.0 for FDP, explaining up to 27.8% and 26.7% of phenotypic variance, respectively. Regarding chilling requirements, notable QTLs were detected on LG1 (S1_9890869) for CH and CP (CH12, CH24, CP24, CP25), with LOD values up to 10.4, especially for CP25, which explained 31.6% of variance, confirming this region as a key regulator of endodormancy release.

For the ‘Currot’ parent in this population, QTLs for BD were primarily located on LG1 and LG7, with a consistent QTL at 46.784 cM on LG1 (S1_9928286) across three years, showing LOD scores of up to 6.9 and explaining 21.9% of variance (Table 2). A strong and stable QTL region for RT was found on LG4 around 54.8 cM, with marker S4_14749428 significantly associated across multiple years (RT12 to RT23), with LOD values up to 7.3 and PEVs near 22%.

For FDP, the most robust signals were located on LG4 at 48.669 cM (S4_12421090), consistently detected across five years (FDP12 to FDP25), reaching LOD scores up to 15.6 and explaining as much as 43.9% of trait variance. Regarding chilling requirement, QTLs were found on LG1 (S1_13528605 and S1_9928286), with CP25 showing the highest effect (LOD 9.1, 28.3% of variance).

#### 2.3.2. ’G × C’ Population

In the ‘Goldrich’ parent, QTLs for BD were mainly located on LG1 (88–93 cM) and LG4 (~47–51 cM) (Table 3). A particularly stable locus on LG1 at 88.221 cM (S1_38997919) was identified across multiple years (BD22, BD25), with the highest LOD value of 20.9 and explaining 45.4% of variance. Similarly, strong QTLs for RT and FDP were concentrated on LG4 at ~40–50 cM, with S4_11831218 being consistently associated with RT (LOD up to 20.2, 44.7% PEV) and FDP (LOD up to 19.3, 43.0% PEV). For chilling requirement, the same region on LG1 (S1_38997919 and S1_39663110) was linked to CH, UCU, and CP, with CP25 presenting a particularly high LOD of 15.0 and explaining 35.7% of variance.

In the ‘Currot’ parent of the ‘G × C’ population, consistent QTLs for BD were also identified on LG4 (55.715 cM, S4_14369731) and LG1 (112.712 cM, S1_40573005), with BD25 reaching a LOD of 23.0 and explaining 48.6% of the trait variance (Table 4). For RT, QTLs were concentrated on LG4 between 51 and 59 cM, with the most significant peak at S4_12863010 (RT13), reaching a LOD of 30.8 and explaining 59.3% of variation. FDP QTLs overlapped with RT loci, particularly at S4_12695425, with consistent effects over several years and a maximum LOD of 19.4 (FDP12), accounting for 43.2% of the variance. The chilling requirement QTLs were again mapped to LG1 (S1_40573005), shared across CH, UCU, and CP, with CP25 showing the strongest signal (LOD 16.0, 37.6% PEV), confirming this region’s involvement in dormancy release mechanisms.

### 2.4. Genes Linked to Major QTLs

Based on the preliminary marker–trait association analyses, which largely coincided with the QTL analysis, although the lead SNPs identified by GWAS and QTL mapping were not always identical because not all GWAS SNPs were incorporated into the final genetic linkage maps, the associated genomic regions were highly consistent between both approaches, as summarized in Table S5. The most significant SNPs for each trait were then classified according to whether they were located in genic or intergenic regions (Table S6). In the ‘G × C’ population, SNPs associated with RT, FDP, BD, and CP were either linked to genes or not linked to genes. Specifically, RT included 18 linked and 12 unlinked SNPs, FDP 17 linked and 13 unlinked, BD 12 linked and 18 unlinked, and CP 12 linked and 18 unlinked.

In the ‘B × C’ population, BD comprised 11 SNPs linked to genes and 19 not linked, whereas CP comprised 16 linked and 14 unlinked SNPs. Regarding BD, *Prupe.1G435100* (COLD REGULATED PROTEIN 27) was found in ‘G × C’. Conversely, in ‘B × C’ BD, *Prupe.1G101500* (histone-lysine N-methyltransferase, ASHR3/SDG) together with *Prupe.1G109800* (DNA cytosine-5 methyltransferase) were identified. As for CP in ‘B × C’, the search revealed *Prupe.1G101500* (histone-lysine N-methyltransferase) as well as *Prupe.1G130400* (RNA polymerase II–associated protein 3).

In terms of RT in ‘G × C’, several candidate genes were identified, including *Prupe.4G190000* (B3 domain transcription factor, VAL1-related), *Prupe.4G176600* (PRA1 family protein 2), *Prupe.4G191600* (vesicle-associated protein), *Prupe.4G234600* (CCCH zinc finger), and *Prupe.4G216900* (dihydrolipoamide succinyltransferase). Finally, for FDP in ‘G × C’, the identified genes comprised *Prupe.4G190000* (B3 transcription factor, VAL1-related), *Prupe.4G234600* (CCCH zinc finger), *Prupe.4G176600* (PRA1 family protein 2), *Prupe.4G154800* (2-oxoglutarate/Fe(II)-dependent dioxygenase), and *Prupe.4G216900* (dihydrolipoamide succinyltransferase).

## 3. Discussion

An accurate phenological characterization is fundamental in breeding programs [25], as this information enables the development of cultivars capable of extending the current market window and thereby meeting increasing consumer demand. However, these traits are mechanistically complex, requiring the establishment of highly specific criteria for data collection [26]. Uncertainty in phenotyping methodologies can introduce background noise due to the lack of consensus on selection standards, a challenge further exacerbated by the wide genetic variability among cultivars, genotype-by-environment interactions, and rapidly changing environmental conditions driven by climate change. In this context, it is noteworthy that the strength and direction of the correlations varied over the years. During the earliest evaluation period (2012–2014), blooming date showed weak or occasionally negative correlations with chilling accumulation. This pattern most likely reflects year-specific climatic conditions, in which winter chilling was not the only limiting factor controlling flowering time. Flowering in apricot results from the sequential fulfillment of chilling and heat requirements [5,27]; therefore, under years with sufficient winter chilling, variation in heat accumulation after dormancy release may become the main determinant of bloom date, reducing the apparent effect of chilling accumulation. In contrast, during the more recent years, characterized by lower winter chill accumulation, the relationship between chilling requirements and blooming date became consistently stronger, particularly when Chill Portions were used. This reduction in winter chill may have enhanced the discriminatory capacity of the Chill Portions model under warmer winter conditions, which are becoming increasingly frequent under current climate change scenarios. Consequently, although some interannual variation was observed, the Chill Portions model provided the strongest overall relationship across the complete eight-year dataset, supporting its suitability for estimating chilling requirements in apricot under contrasting climatic conditions [28].

The construction of genetic linkage maps is a suitable method for identifying genomic regions that control both qualitative and quantitative traits [29]. In previous work, linkage maps were constructed by combining SSR and SNP markers in apricot, using 87 markers in the ‘B × C’ population and 89 markers in the ‘G × C’ population [30]. The results revealed high variability and segregation in both populations, which were evaluated over three consecutive years. In that study, QTLs associated with BD, RT, and FDP were consistently detected across years in all LGs except LG8. Among them, major QTLs for BD, RT, and FDP were identified on LG4, particularly in the ‘G × C’ population. In the present study, the development of high-resolution linkage maps enabled the precise localization of QTLs for key phenological traits in apricot. For example, multi-locus QTLs for BD were detected on LG1 and LG7 in the ‘B × C’ population, and on LG1 and LG4 in the ‘G × C’ population. Additionally, stable QTLs were identified on LG1 for CR, and on LG4 for both RT and FDP. Consistent with our findings, several QTLs associated with flowering and maturity dates demonstrated remarkable stability across years of evaluation, suggesting that their expression was largely unaffected by interannual climatic variation [31].

Comparative studies across *Prunus* species have revealed conserved genomic regions associated with key agronomic traits, such as flowering time and the interval between bloom and harvest [32]. In peach, QTLs for both flowering and fruit development have been localized to LG4 and LG6 [33], while in sweet cherry, QTLs for chilling requirement and flowering date have also been detected on LG4 [34]. These concordant results suggest that common chromosomal regions are central to the regulation of phenological traits within the genus.

Despite phenotypic evaluations being conducted in two non-consecutive periods (2012–2014 and 2021–2025), the major QTLs identified for blooming date, chilling requirement, fruit development period, and ripening time remained remarkably stable across years. This indicates that although environmental conditions—including the lower winter chill accumulation observed during the most recent seasons—and the increase in tree age may have influenced phenotypic expression, they did not substantially alter the principal genomic regions controlling these traits. The stability of these QTLs across contrasting climatic conditions highlights their robustness and reinforces their potential value for marker-assisted breeding aimed at improving adaptation to climate change [35].

In addition, numerous candidate genes have been linked to flowering control, including transcription factors and regulators of hormonal or light perception pathways [36]. Genes such as *APETALA1* and members of the *MADS-box* family [37], *SOC1* [38], and *TFL1* [39] contribute to floral transition and meristem identity, while photoreceptors like *PHY A*, *PHY B* and *PHY E* mediate environmental cues [40]. *DAM* genes, particularly *DAM5* and *DAM6*, have also been implicated in dormancy release (i.e., chilling requirements determination) and flowering regulation [41,42], reinforcing the idea of a highly conserved yet complex genetic network across *Prunus* species.

Consistently, in the low-chilling sweet cherry Spanish cultivar ‘Cristobalina’, a QTL on LG1 co-localized with *DAM* genes, echoing results from peach and supporting their role as major determinants of chilling requirement. Within these regions, additional candidates involved in chromatin remodeling (*ARP4*, *EMF2*, *PIE1*) and gibberellin metabolism (*KS*, *GA2ox*) were also detected [43], suggesting an interplay between epigenetic regulation and hormonal pathways in dormancy release. Similarly, in almond, genes associated with gibberellin metabolism have also been recently identified [44]. Moreover, studies in peach have highlighted genes such as *ppLFL* (a *LEAFY/FLORICAULA* homolog) as potential regulators of floral induction [45].

In agreement with these studies, we identified *Prupe.1G435100*, encoding *COLD REGULATED PROTEIN 27*, as a strong candidate for BD, consistent with the role of canonical COR/CBF proteins in cold response and dormancy regulation [46,47,48]. In addition, epigenetic regulators emerged as key players: *Prupe.1G101500* (histone-lysine N-methyltransferase, ASHR3/SDG), which may modulate dormancy genes through histone methylation, and *Prupe.1G109800* (DNA cytosine-5 methyltransferase), implicated in DNA methylation processes underlying vernalization and dormancy control [49]. Notably, *Prupe.1G101500* was also detected for chilling requirement, further supporting the pivotal role of histone methylation in the regulation of dormancy transitions.

Integration of the FDP and RT results revealed a convergent set of candidate genes, emphasizing the common genetic determinants that regulate these interrelated phenological processes. Among them, *Prupe.4G190000* (a B3-domain transcription factor related to VAL1) and *Prupe.4G234600* (a CCCH-type zinc finger protein) show medium to high plausibility for contributing to developmental timing in both FDP and RT [50,51,52,53]. Vesicular trafficking components, particularly *Prupe.4G176600* (PRA1 family protein 2), suggest that intracellular transport mechanisms may contribute to hormone distribution or cell wall materials critical during both fruit growth and the ripening phase. Additional genes such as *Prupe.4G216900* (dihydrolipoamide succinyltransferase) appear with moderate relevance, likely reflecting more general metabolic or bioenergetic roles rather than ripening-specific regulation. Together, these results indicate that while FDP and RT are phenologically distinct, much of their genetic basis overlaps, particularly in transcriptional regulators.

However, for peach, a maturity date locus (qMD4.1) on LG4 was fine-mapped to a ~220 kb region, where two NAC transcription factors; *ppa007577m* and *ppa008301m*, were identified as strong candidates. Among these, *ppa008301m* was found to carry a 9 bp in-frame insertion in its last exon that co-segregated with early maturity alleles in both studied populations [54]. Similarly, ANAC072 has been proposed as a candidate gene within LG4 QTLs for maturity date and mapped traits in peach germplasm [55]. These NAC genes are especially interesting because NAC family transcription factors are known to regulate diverse aspects of fruit ripening, including cell wall modification, ethylene signaling, and developmental timing [56]. The repeated identification of NAC transcription factors as candidate genes, particularly those co-localized within major QTLs that explain a substantial proportion of phenotypic variance, strongly supports their role as central regulators of fruit maturation and developmental transitions. In line with our findings—where additional transcriptional regulators such as B3 domain proteins and CCCH zinc finger proteins were also implicated in both FDP and RT—it is plausible that conserved or orthologous regulatory pathways operate across *Prunus* species. Notably, the presence of NAC genes within the corresponding QTL region has also been confirmed in the available apricot genome (NCBI), specifically at loci *CURHAP_LOCUS27365* and *CURHAP_LOCUS27368*, further reinforcing their candidacy in the genetic control of these phenological traits.

Future work should focus on validating the expression profiles of these candidate genes across developmental stages and assessing whether polymorphisms (such as frame insertions/deletions or regulatory variants) in these transcription factors correlate with phenotypic variation in FDP/RT in apricot. Notably, many of the significant SNPs detected are located in intergenic regions, which may play a crucial role as they could correspond to promoter sequences or other regulatory elements controlling genes involved in these phenological traits. Therefore, further investigations are required to unravel the regulatory mechanisms underlying these regions of interest and their contribution to phenological variation.

## 4. Materials and Methods

### 4.1. Plant Material and Experimental Site

The plant material included two F1 apricot populations: ‘Bergeron’ × ‘Currot’ (‘B × C’, n = 134) and ‘Goldrich’ × ‘Currot’ (‘G × C’, n = 159). Both populations were established in 2009 at the experimental orchard of CEBAS-CSIC, located in Cieza-Calasparra, Murcia, Spain (37° N, 1° W, 450 m above sea level). ‘Bergeron’ is a self-compatible French cultivar characterized by a high chilling requirement (approximately 65 Chill Portions) [5], late blooming, and late fruit maturity. In contrast, ‘Goldrich’ is a self-incompatible cultivar with a medium-high chilling requirement (around 58 Chill Portions) [57], mid-to-late blooming, and mid-season fruit maturity. ‘Currot’, a traditional Spanish cultivar serving as the common male parent in both crosses, has a low chilling requirement (approximately 38 Chill Portions) [58], and is noted for its very early blooming and very early fruit ripening [59].

### 4.2. Phenotyping and Analysis of Phenological Traits

Phenotyping encompassed the assessment of chilling requirements as well as several key phenological traits, including blooming date (BD), fruit development period (FDP), and ripening time (RT). These measurements were conducted in both F1 populations across non-consecutive eight growing seasons (2012, 2013, 2014, 2021, 2022, 2023, 2024, and 2025) (Table S1). Since all years fall within the 2000s, phenotypic records are identified using the last two digits of the year. Blooming and ripening assessments were carried out under field conditions and expressed in Julian days (day of the year). BD was recorded when approximately 50% of the flowers were open (F50) [60], whereas RT was determined based on the attainment of commercial maturity, identified by the characteristic skin color change and fruit firmness. FDP was calculated as the number of days between blooming and ripening. Chilling requirements were estimated following a recently published standardized workflow for determining agroclimatic requirements in biparental populations with a large number of individuals [61]. Since the development and validation of the Partial Least Squares (PLS)-based workflow were previously reported as a dedicated methodological study, only the chilling requirement values obtained from that validated approach were used in the present work for the subsequent genetic analyses. The three most commonly used models in fruit research also showing increasing complexity in the mathematical structure of the model were applied: chill hours [62], Utah chill units [63], and chill portions [64,65,66] (Table S2).

### 4.3. Marker-Trait Association (GWAS) and QTL Analyses

Associations between SNPs and phenotypic traits were analyzed using the software TASSEL v5 [67]. A General Linear Model (GLM) was applied, integrating phenotypic measurements with genotypic data, and accounting for population structure through principal component analysis (PCA) (Tables S3 and S4). The genome-wide significance threshold was determined using the Bonferroni correction for multiple testing. To visualize the genomic distribution and significance of marker-trait associations, Manhattan plots were generated for the most informative traits across different years.

For QTL detection, both parametric and non-parametric mapping strategies were employed using MAPQTL v7. Interval mapping was conducted to estimate the genomic positions of putative QTLs, along with their confidence intervals. Additionally, the Kruskal–Wallis test—a distribution-free method—was used to confirm marker-trait associations independently of normality assumptions. The significance of QTL peaks was determined by calculating LOD (logarithm of odds) score thresholds, based on 1000 permutation tests performed with the built-in “Permutation Test” function. Genetic maps for both populations were developed in the previous research work [68]. Briefly, GBS was previously performed for the two F1 populations (‘B × C’, n = 134; ‘G × C’, n = 159). Libraries were generated using the ApeKI restriction enzyme and sequenced on Illumina NextSeq 500/550 and NovaSeq 6000 platforms (150 bp paired-end), producing approximately 450 million reads (≈1.5 million reads per sample). After quality filtering, adapter trimming, alignment to the *Prunus armeniaca* reference genome, and SNP calling, stringent filtering criteria were applied to remove low-quality and highly missing markers, resulting in 45,704 and 52,166 high-confidence SNPs for the ‘B × C’ and ‘G × C’ populations, respectively. These SNP datasets were subsequently used to construct the high-density linkage maps and for the GWAS and QTL analyses performed in the present study.

### 4.4. Identification of Candidate Genes Associated with Major QTLs

Candidate gene identification was carried out focusing on the main QTL regions associated with key phenological traits: BD, CR, FDP, and RT. As ‘Currot’ is the common male parent in both F1 populations and its reference genome is available in the NCBI database [69], this genome was used to locate the genomic regions associated with the identified QTLs. However, because the current ‘Currot’ genome assembly lacks functional gene annotation, the sequences surrounding each significant SNP were extracted and aligned against the functionally annotated *Prunus persica* v2.1 reference genome using the Phytozome platform (https://phytozome-next.jgi.doe.gov/ (accessed on 3 May 2026)). Owing to the high degree of synteny between peach and apricot genomes, this approach enabled the identification and functional annotation of candidate genes located within the QTL intervals. The correspondence between the *Prunus persica* gene identifiers and their homologous loci in the ‘Currot’ genome is provided in the supplementary table (Table S6).

### 4.5. Data Analysis

All analyses and figure preparation related to genetic linkage maps and QTL visualization were performed using the R software environment (version 4.3.2; RStudio team, Boston, MA, USA). Genetic linkage maps were rendered using the packages LinkageMapView, which also served for the graphical representation of QTL results. For the calculation of chill requirements, the chillR package [70] for R was used using the workflow modified [61]. In addition, ggplot2 was employed to generate violin plots.

## 5. Conclusions

This study provides a comprehensive dissection of the genetic architecture underlying apricot phenology and chilling requirements. By combining multi-year phenotypic evaluations with GBS-derived high-density genetic maps, we identified robust and stable QTLs on LG1 for blooming date and chilling requirement, and on LG4 for fruit development period and ripening time. These loci explained a substantial proportion of trait variance, confirming their central role in the regulation of dormancy release and growth developmental timing. Epigenetic control elements, cold-regulated proteins, and key transcription factors emerged from candidate gene analysis as likely contributors to the genetic regulation of phenology, linking genotype to phenotype.

The integration of marker–trait association and linkage mapping approaches enhanced the resolution and reliability of trait–marker associations, establishing a strong foundation for developing molecular markers in apricot. These findings not only contribute to understanding the genetic mechanisms of dormancy and phenology in *Prunus* but also provide evidence that could be used to guide marker-assisted selection strategies. Ultimately, this work supports the development of new apricot cultivars with desired chilling requirements and flowering times, better adapted to the challenges of climate change and capable of extending the commercial harvest season.

## Figures and Tables

**Table 1:** QTLs identified in the ‘Bergeron’ parent from the ‘B × C’ cross for phenology traits.

‘Bergeron’ (‘B × C’) Traits ^a^	LG	Position (cM)	Locus	LOD ^b^	K ^c^	PEV ^d^
CH12	1	21.014	S1_9890869	5.3	24.5	17.6
CH24	1	21.014	S1_9890869	5.1	24.3	17.1
CP24	1	21.014	S1_9890869	5.2	22.6	17.3
CP25	1	21.014	S1_9890869	10.4	38.2	31.6
BD12	7	32.071	S7_15878965	6.5	27.1	19.9
BD13	1	98.262	S1_42896153	5.4	19.4	17.0
BD14	7	38.592	S7_17185342	5.9	25.1	18.5
BD21	1	21.014	S1_9890869	7.0	29.0	21.6
BD22	4	59.141	S4_22646518	5.9	15.7	18.9
BD23	7	40.827	S7_18085942	5.4	17.8	17.0
BD24	1	21.014	S1_9890869	6.4	25.6	20.1
BD25	1	21.014	S1_9890869	7.3	29.2	23.1
FDP12	4	48.887	S4_12380084	9.0	37.6	26.7
FDP13	4	49.376	S4_12383019	5.4	22.6	17.0
FDP14	4	48.800	S4_12377040	8.6	33.8	25.6
FDP21	4	41.021	S4_10082360	5.5	17.4	17.8
FDP22	4	43.186	S4_11065125	13.3	42.8	39.1
FDP23	4	41.021	S4_10082360	7.7	26.3	23.6
FDP24	4	38.312	S4_9749496	7.5	17.7	23.3
FDP25	4	43.186	S4_11065125	9.2	35.8	28.3
RT12	4	48.887	S4_12380084	7.7	30.5	23.1
RT13	4	48.887	S4_12380084	7.9	32.3	23.6
RT14	4	48.800	S4_12377040	9.4	30.9	27.8
RT21	4	48.887	S4_12380084	6.3	24.0	20.1
RT22	4	39.977	S4_9840503	8.7	20.2	27.2
RT23	4	48.887	S4_12380084	7.2	29.0	22.5
RT24	4	48.887	S4_12380084	6.7	27.6	20.9
RT25	4	48.887	S4_12380084	8.0	29.5	24.2

^a^ CH: chill hours, UCU: Utah chill units, CP: chill portions, BD: blooming date, FDP: fruit development period, RT: ripening time, ^b^ ‘LOD’ represents the statistic obtained from the interval mapping test. ^c^ ‘K’ refers to the statistic derived from the Kruskal–Wallis test. ^d^ ‘PEV’ indicates the percentage of phenotypic variation explained by the marker.

**Table 2:** QTLs identified in the ‘Currot’ parent from the ‘B × C’ cross for phenology traits.

‘Currot’ (‘B × C’) Traits ^a^	LG	Position (cM)	Locus	LOD ^b^	K ^c^	PEV ^d^
CH12	1	52.814	S1_13528605	5.9	24.9	19.5
CH24	1	52.814	S1_13528605	6.3	26.7	20.5
CP24	1	52.814	S1_13528605	6.6	25.9	21.3
CP25	1	46.784	S1_9928286	9.1	35.1	28.3
BD12	7	42.204	S7_16347087	7.2	14.1	21.9
BD13	7	39.646	S7_15373247	4.9	12.8	15.4
BD14	7	39.646	S7_15373247	5.6	26.8	17.5
BD21	1	46.784	S1_9928286	6.9	27.2	21.5
BD22	4	58.528	S4_16316561	5.4	19.4	17.5
BD24	1	46.784	S1_9928286	6.1	22.4	19.3
BD25	1	46.784	S1_9928286	6.9	26.8	21.9
FDP12	4	48.669	S4_12421090	11.2	35.1	31.9
FDP13	4	48.669	S4_12421090	8.4	20.8	25.1
FDP14	4	48.669	S4_12421090	9.0	35.1	26.9
FDP21	4	43.277	S4_10232868	6.1	14.0	19.7
FDP22	4	48.669	S4_12421090	15.6	18.4	43.9
FDP23	4	43.277	S4_10232868	8.0	29.3	24.5
FDP24	4	48.669	S4_12421090	9.2	17.0	27.9
FDP25	4	48.669	S4_12421090	11.3	15.0	33.4
RT12	4	54.858	S4_14749428	6.8	21.7	20.8
RT13	4	54.858	S4_14749428	6.9	25.7	21.1
RT14	4	54.795	S4_14709453	7.3	14.5	22.3
RT21	4	54.858	S4_14749428	5.6	12.3	18.0
RT22	4	54.858	S4_14749428	7.0	22.2	22.6
RT23	4	55.573	S4_14759605	6.1	12.4	19.3
RT24	4	54.757	S4_14564191	7.0	18.9	21.9
RT25	4	54.757	S4_14564191	6.1	20.2	18.9

^a^ CH: chill hours, CP: chill portions, BD: blooming date, FDP: fruit development period, RT: ripening time. ^b^ ‘LOD’ represents the statistic obtained from the interval mapping test. ^c^ ‘K’ refers to the statistic derived from the Kruskal–Wallis test. ^d^ ‘PEV’ indicates the percentage of phenotypic variation explained by the marker.

**Table 3:** QTLs identified in the ‘Goldrich’ parent from the ‘G × C’ cross for phenology traits.

‘Goldrich’ (‘G × C’) Traits ^a^	LG	Position (cM)	Locus	LOD ^b^	K ^c^	PEV ^d^
CH12	1	92.558	S1_39509319	6.5	27.2	17.5
CH25	1	88.221	S1_38997919	8.0	54.4	21.0
UCU24	1	92.834	S1_39663110	10.1	44.7	25.7
UCU25	1	92.834	S1_39663110	9.9	41.1	25.3
CP25	1	88.221	S1_38997919	15.0	55.5	35.7
BD12	4	50.832	S4_16824752	9.4	33.2	24.0
BD13	4	47.832	S4_15055783	10.2	38.3	25.5
BD14	4	47.832	S4_15055783	14.4	64.3	34.0
BD21	1	87.715	S1_38992466	9.4	41.2	23.8
BD22	1	88.221	S1_38997919	13.8	46.7	33.0
BD23	1	92.834	S1_39663110	11.4	43.0	28.2
BD24	1	92.834	S1_39663110	20.5	69.6	45.0
BD25	1	88.221	S1_38997919	20.9	67.3	45.4
FDP12	4	40.228	S4_11831218	19.3	60.6	43.0
FDP13	4	40.228	S4_11831218	10.3	27.6	26.0
FDP14	4	40.228	S4_11831218	5.6	19.8	15.0
FDP21	4	40.228	S4_11831218	11.7	43.9	29.9
FDP22	4	40.228	S4_11831218	11.4	28.1	28.6
FDP23	4	40.228	S4_11831218	7.5	27.9	19.9
FDP24	4	40.228	S4_11831218	8.9	28.3	23.1
FDP25	4	40.228	S4_11831218	8.6	38.5	22.1
RT12	4	50.832	S4_16824752	23.8	43.6	49.7
RT13	4	47.832	S4_15055783	28.0	58.0	55.8
RT14	4	50.832	S4_16824752	23.2	59.8	49.1
RT21	4	47.832	S4_15055783	18.6	52.1	43.3
RT22	4	40.228	S4_11831218	20.2	65.9	44.7
RT23	4	47.832	S4_15055783	17.4	54.8	40.3
RT24	4	40.228	S4_11831218	18.4	60.1	41.5
RT25	4	40.228	S4_11831218	16.8	61.1	38.5

^a^ CH: chill hours, UCU: Utah chill units, CP: chill portions, BD: blooming date, FDP: fruit development period, RT: ripening time. ^b^ ‘LOD’ represents the statistic obtained from the interval mapping test. ^c^ ‘K’ refers to the statistic derived from the Kruskal–Wallis test. ^d^ ‘PEV’ indicates the percentage of phenotypic variation explained by the marker.

**Table 4:** QTLs identified in the ‘Currot’ parent from the ‘G × C’ cross for phenology traits.

‘Currot’ (‘G × C’) Traits ^a^	LG	Position (cM)	Locus	LOD ^b^	K ^c^	PEV ^d^
CH12	1	113.479	S1_40650682	6.5	21.1	17.4
CH25	1	111.436	S1_40331683	8.7	48.1	22.7
UCU24	1	112.712	S1_40573005	12.0	37.2	29.7
UCU25	1	112.712	S1_40573005	10.4	32.0	26.4
CP25	1	112.712	S1_40573005	16.0	46.6	37.6
BD12	4	55.715	S4_14369731	9.5	26.5	24.2
BD13	4	55.715	S4_14369731	10.1	23.2	25.4
BD14	4	55.715	S4_14369731	14.1	36.3	33.6
BD21	1	112.712	S1_40573005	8.0	29.4	20.8
BD22	1	112.712	S1_40573005	12.1	34.0	29.8
BD23	1	112.712	S1_40573005	12.0	35.5	29.3
BD24	1	112.712	S1_40573005	24.0	52.6	50.4
BD25	1	112.712	S1_40573005	23.0	55.8	48.6
FDP12	4	51.955	S4_12695425	19.4	55.5	43.2
FDP13	4	51.955	S4_12695425	11.4	45.2	28.3
FDP14	4	59.340	S4_14816248	6.4	25.3	17.1
FDP21	4	51.955	S4_12695425	11.7	21.7	30.1
FDP22	4	51.955	S4_12695425	12.5	38.2	30.8
FDP23	4	66.056	S4_18156937	9.3	20.3	24.0
FDP24	4	51.955	S4_12695425	10.3	33.9	26.1
FDP25	4	51.240	S4_12611878	13.0	42.1	31.3
RT12	4	59.340	S4_14816248	22.6	42.5	48.0
RT13	4	53.107	S4_12863010	30.8	50.1	59.3
RT14	4	59.340	S4_14816248	23.2	25.2	49.2
RT21	4	59.340	S4_14816248	18.1	22.0	42.4
RT22	4	51.955	S4_12695425	19.3	37.5	43.2
RT23	4	59.340	S4_14816248	17.4	13.4	40.4
RT24	4	59.340	S4_14816248	20.0	33.0	44.2
RT25	4	51.240	S4_12611878	17.4	26.8	39.5

^a^ BD: blooming date, FDP: fruit development period, RT: ripening time, CH: chill hours, UCU: Utah chill units, CP: chill portions. ^b^ ‘LOD’ represents the statistic obtained from the interval mapping test. ^c^ ‘K’ refers to the statistic derived from the Kruskal–Wallis test. ^d^ ‘PEV’ indicates the percentage of phenotypic variation explained by the marker.

## Data Availability

The datasets generated and/or analyzed during the current study are included in this published article and its Supplementary Information files. Additional information is available from the corresponding author upon reasonable request.

## Supplementary Materials

The following supporting information can be downloaded at: https://www.mdpi.com/article/10.3390/ijms27146264/s1.
