# Supplementary material for: Genetic Dissection of Adaptation Traits in Apricot Through GWAS and QTL Analyses
Source: Int J Mol Sci. 2026 Jul 14;27(14):6264. doi: 10.3390/ijms27146264 (PMC13410052; doi:10.3390/ijms27146264)
Supplement: Supplementary file 1 [file ijms-27-06264-s001.zip › ijms-4401808 R1 Figure S1.pdf]

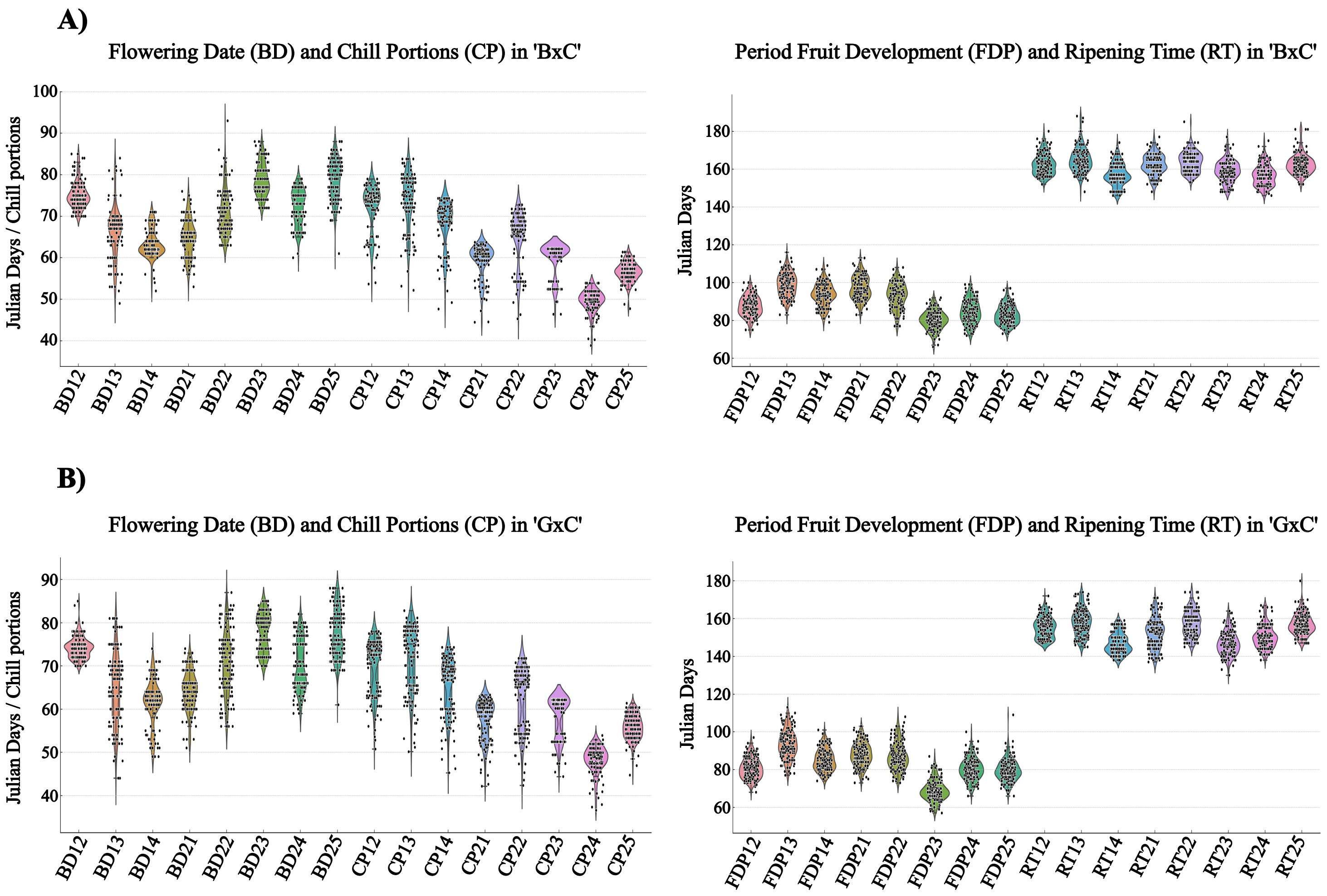
A)
Flowering Date (BD) and Chill Portions (CP) in 'BxC'
Julian Days / Chill portions
BD12 BD13 BD14 BD21 BD22 BD23 BD24 BD25 CP12 CP13 CP14 CP21 CP22 CP23 CP24 CP25
Period Fruit Development (FDP) and Ripening Time (RT) in 'BxC'
Julian Days
FDP12 FDP13 FDP14 FDP21 FDP22 FDP23 FDP24 FDP25 RT12 RT13 RT14 RT21 RT22 RT23 RT24 RT25
B)
Flowering Date (BD) and Chill Portions (CP) in 'GxC'
Julian Days / Chill portions
BD12 BD13 BD14 BD21 BD22 BD23 BD24 BD25 CP12 CP13 CP14 CP21 CP22 CP23 CP24 CP25
Period Fruit Development (FDP) and Ripening Time (RT) in 'GxC'
Julian Days
FDP12 FDP13 FDP14 FDP21 FDP22 FDP23 FDP24 FDP25 RT12 RT13 RT14 RT21 RT22 RT23 RT24 RT25
