# Supplementary material for: Genetic Dissection of Adaptation Traits in Apricot Through GWAS and QTL Analyses
Source: Int J Mol Sci. 2026 Jul 14;27(14):6264. doi: 10.3390/ijms27146264 (PMC13410052; doi:10.3390/ijms27146264)
Supplement: Supplementary file 1 [file ijms-27-06264-s001.zip › ijms-4401808 R1 Figure S2.pdf]

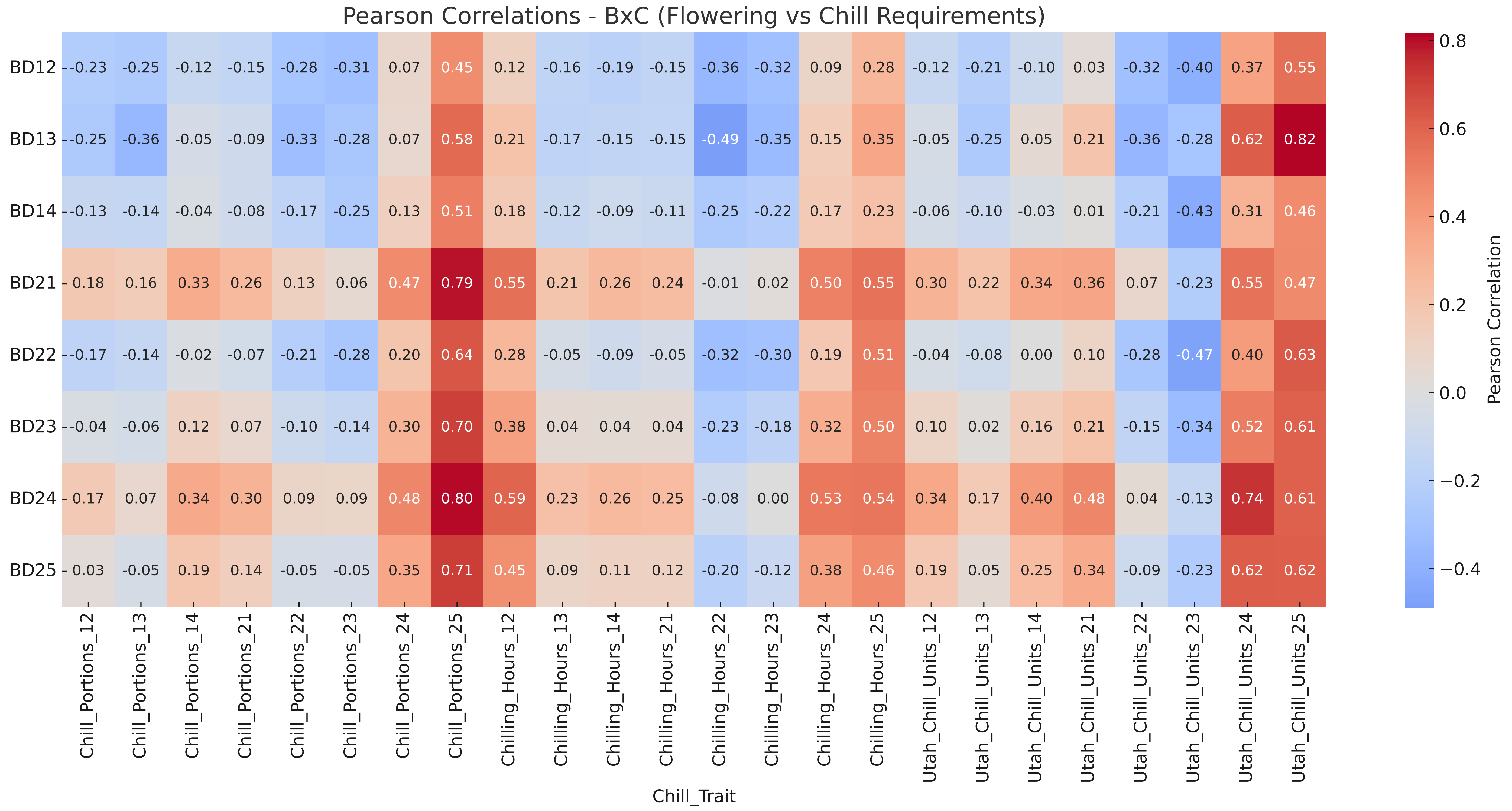
Pearson Correlations - BxC (Flowering vs Chill Requirements)
BD12 -0.23 -0.25 -0.12 -0.15 -0.28 -0.31 0.07 0.45 0.12 -0.16 -0.19 -0.15 -0.36 -0.32 0.09 0.28 -0.12 -0.21 -0.10 0.03 -0.32 -0.40 0.37 0.55
BD13 -0.25 -0.36 -0.05 -0.09 -0.33 -0.28 0.07 0.58 0.21 -0.17 -0.15 -0.15 -0.49 -0.35 0.15 0.35 -0.05 -0.25 0.05 0.21 -0.36 -0.28 0.62 0.82
BD14 -0.13 -0.14 -0.04 -0.08 -0.17 -0.25 0.13 0.51 0.18 -0.12 -0.09 -0.11 -0.25 -0.22 0.17 0.23 -0.06 -0.10 -0.03 0.01 -0.21 -0.43 0.31 0.46
BD21 0.18 0.16 0.33 0.26 0.13 0.06 0.47 0.79 0.55 0.21 0.26 0.24 -0.01 0.02 0.50 0.55 0.30 0.22 0.34 0.36 0.07 -0.23 0.55 0.47
BD22 -0.17 -0.14 -0.02 -0.07 -0.21 -0.28 0.20 0.64 0.28 -0.05 -0.09 -0.05 -0.32 -0.30 0.19 0.51 -0.04 -0.08 0.00 0.10 -0.28 -0.47 0.40 0.63
BD23 -0.04 -0.06 0.12 0.07 -0.10 -0.14 0.30 0.70 0.38 0.04 0.04 0.04 -0.23 -0.18 0.32 0.50 0.10 0.02 0.16 0.21 -0.15 -0.34 0.52 0.61
BD24 0.17 0.07 0.34 0.30 0.09 0.09 0.48 0.80 0.59 0.23 0.26 0.25 -0.08 0.00 0.53 0.54 0.34 0.17 0.40 0.48 0.04 -0.13 0.74 0.61
BD25 0.03 -0.05 0.19 0.14 -0.05 -0.05 0.35 0.71 0.45 0.09 0.11 0.12 -0.20 -0.12 0.38 0.46 0.19 0.05 0.25 0.34 -0.09 -0.23 0.62 0.62
Chill_Portions_12
Chill_Portions_13
Chill_Portions_14
Chill_Portions_21
Chill_Portions_22
Chill_Portions_23
Chill_Portions_24
Chill_Portions_25
Chilling_Hours_12
Chilling_Hours_13
Chilling_Hours_14
Chilling_Hours_21
Chilling_Hours_22
Chilling_Hours_23
Chilling_Hours_24
Chilling_Hours_25
Utah_Chill_Units_12
Utah_Chill_Units_13
Utah_Chill_Units_14
Utah_Chill_Units_21
Utah_Chill_Units_22
Utah_Chill_Units_23
Utah_Chill_Units_24
Utah_Chill_Units_25
Chill_Trait
Pearson Correlation
0.8
0.6
0.4
0.2
0.0
−0.2
−0.4

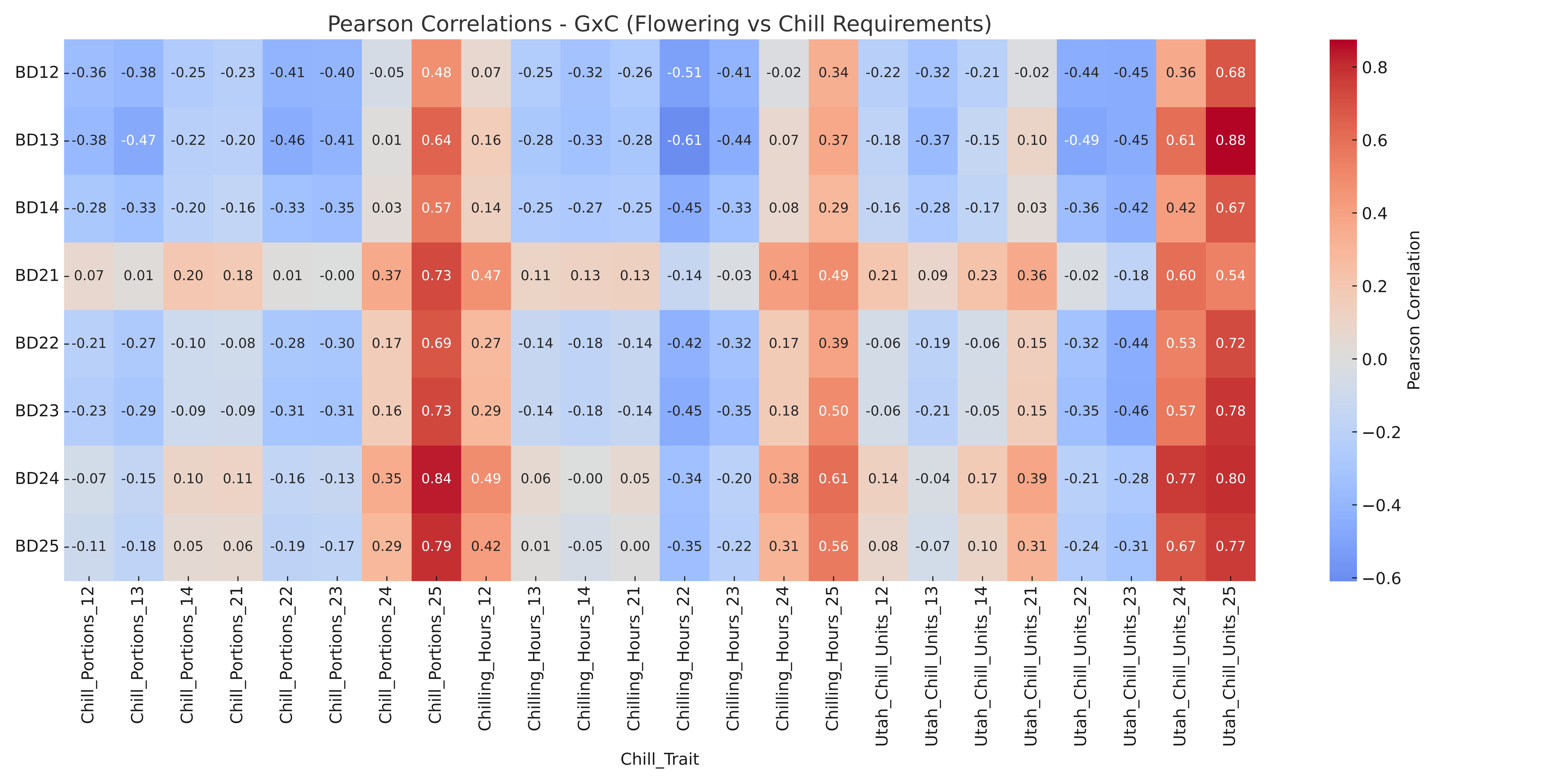
Pearson Correlations - GxC (Flowering vs Chill Requirements)
BD12 -0.36 -0.38 -0.25 -0.23 -0.41 -0.40 -0.05 0.48 0.07 -0.25 -0.32 -0.26 -0.51 -0.41 -0.02 0.34 -0.22 -0.32 -0.21 -0.02 -0.44 -0.45 0.36 0.68
BD13 -0.38 -0.47 -0.22 -0.20 -0.46 -0.41 0.01 0.64 0.16 -0.28 -0.33 -0.28 -0.61 -0.44 0.07 0.37 -0.18 -0.37 -0.15 0.10 -0.49 -0.45 0.61 0.88
BD14 -0.28 -0.33 -0.20 -0.16 -0.33 -0.35 0.03 0.57 0.14 -0.25 -0.27 -0.25 -0.45 -0.33 0.08 0.29 -0.16 -0.28 -0.17 0.03 -0.36 -0.42 0.42 0.67
BD21 0.07 0.01 0.20 0.18 0.01 -0.00 0.37 0.73 0.47 0.11 0.13 0.13 -0.14 -0.03 0.41 0.49 0.21 0.09 0.23 0.36 -0.02 -0.18 0.60 0.54
BD22 -0.21 -0.27 -0.10 -0.08 -0.28 -0.30 0.17 0.69 0.27 -0.14 -0.18 -0.14 -0.42 -0.32 0.17 0.39 -0.06 -0.19 -0.06 0.15 -0.32 -0.44 0.53 0.72
BD23 -0.23 -0.29 -0.09 -0.09 -0.31 -0.31 0.16 0.73 0.29 -0.14 -0.18 -0.14 -0.45 -0.35 0.18 0.50 -0.06 -0.21 -0.05 0.15 -0.35 -0.46 0.57 0.78
BD24 -0.07 -0.15 0.10 0.11 -0.16 -0.13 0.35 0.84 0.49 0.06 -0.00 0.05 -0.34 -0.20 0.38 0.61 0.14 -0.04 0.17 0.39 -0.21 -0.28 0.77 0.80
BD25 -0.11 -0.18 0.05 0.06 -0.19 -0.17 0.29 0.79 0.42 0.01 -0.05 0.00 -0.35 -0.22 0.31 0.56 0.08 -0.07 0.10 0.31 -0.24 -0.31 0.67 0.77
Chill_Portions_12
Chill_Portions_13
Chill_Portions_14
Chill_Portions_21
Chill_Portions_22
Chill_Portions_23
Chill_Portions_24
Chill_Portions_25
Chilling_Hours_12
Chilling_Hours_13
Chilling_Hours_14
Chilling_Hours_21
Chilling_Hours_22
Chilling_Hours_23
Chilling_Hours_24
Chilling_Hours_25
Utah_Chill_Units_12
Utah_Chill_Units_13
Utah_Chill_Units_14
Utah_Chill_Units_21
Utah_Chill_Units_22
Utah_Chill_Units_23
Utah_Chill_Units_24
Utah_Chill_Units_25
Chill_Trait
Pearson Correlation
0.8
0.6
0.4
0.2
0.0
−0.2
−0.4
−0.6
