# Supplementary material for: Genetic Dissection of Adaptation Traits in Apricot Through GWAS and QTL Analyses
Source: Int J Mol Sci. 2026 Jul 14;27(14):6264. doi: 10.3390/ijms27146264 (PMC13410052; doi:10.3390/ijms27146264)
Supplement: Supplementary file 1 [file ijms-27-06264-s001.zip › ijms-4401808 R1 Table S5.pdf]

International Journal of
Molecular Sciences

MDPI

Table S5.1. Correspondence between significant QTLs and their associated GWAS SNPs and association statistics (-$\log_{10}$(P-value) and R^2) in the 'B×C' and 'G×C' populations.

('BxC') Traits[a]	LG	Locus	LOD[b]	K[c]	PEV[d]	-$\log_{10}$(p-value)[e]	R^{2e}
CH12	1	S1_9890869	5.3	24.5	17.6	3.3×10^{-5}	0.15
CH24	1	S1_9890869	5.1	24.3	17.1	8.9×10^{-6}	0.16
CP24	1	S1_9890869	5.2	22.6	17.3	6.7×10^{-6}	0.17
CP25	1	S1_9890869	10.4	38.2	31.6	1.3×10^{-6}	0.16
BD12	7	S7_15878965	6.5	27.1	19.9	2.2×10^{-2}	0.06
BD13	1	S1_42896153	5.4	19.4	17.0	2.5×10^{-2}	0.04
BD14	7	S7_17185342	5.9	25.1	18.5	2.3×10^{-6}	0.15
BD21	1	S1_9890869	7.0	29.0	21.6	1.7×10^{-4}	0.10
BD22	4	S4_22034522	5.5	16.2	17.6	1.6×10^{-7}	0.19
BD23	7	S7_18085942	5.4	17.8	17.0	2.9×10^{-4}	0.10
BD24	1	S1_9890869	6.4	25.6	20.1	3.7×10^{-5}	0.13
BD25	1	S1_9890869	7.3	29.2	23.1	1.4×10^{-5}	0.13
FDP12	4	S4_12380084	9.0	37.6	26.7	1.9×10^{-8}	0.25
FDP13	4	S4_12383019	5.4	22.6	17.0	1.0×10^{-6}	0.21
FDP14	4	S4_12377040	8.6	33.8	25.6	2.4×10^{-7}	0.22
FDP21	4	S4_10082360	5.5	17.4	17.8	2.6×10^{-4}	0.14
FDP22	4	S4_11065125	13.3	42.8	39.1	1.7×10^{-12}	0.36
FDP23	4	S4_10082360	7.7	26.3	23.6	1.9×10^{-6}	0.20
FDP24	4	S4_9749496	7.5	17.7	23.3	7.8×10^{-6}	0.20
FDP25	4	S4_11065125	9.2	35.8	28.3	8.7×10^{-9}	0.26
RT12	4	S4_12380084	7.7	30.5	23.1	5.2×10^{-9}	0.24
RT13	4	S4_12380084	7.9	32.3	23.6	3.4×10^{-9}	0.22
RT14	4	S4_12377040	9.4	30.9	27.8	5.0×10^{-8}	0.22
RT21	4	S4_12380084	6.3	24.0	20.1	1.1×10^{-4}	0.13
RT22	4	S4_9840503	8.7	20.2	27.2	6.2×10^{-10}	0.27
RT23	4	S4_12380084	7.2	29.0	22.5	1.7×10^{-6}	0.18
RT24	4	S4_12380084	6.7	27.6	20.9	2.8×10^{-7}	0.22
RT25	4	S4_12380084	8.0	29.5	24.2	1.1×10^{-6}	0.17

[a] CH: chill hours, UCU: Utah chill units, CP: chill portions, BD: blooming date, FDP: fruit development period, RT: ripening time, [b] 'LOD' represents the statistic obtained from the interval mapping test. [c] 'K' refers to the statistic derived from the Kruskal-Wallis test. [d] 'PEV' indicates the percentage of phenotypic variation explained by the marker. [e] Parameters used for the marker–trait association (GWAS) analysis.

Table S5.2. Correspondence between significant QTLs and their associated GWAS SNPs and association statistics (-log_{10}(P-value) and R^2) in the 'B×C' and 'G×C' populations.

('GxC') Traits[a]	LG	Locus	LOD[b]	K[c]	PEV[d]	-log_{10}(p-value)[e]	R^{2e}
CH12	1	S1_39509319	6.5	27.2	17.5	2.0×10^{-6}	0.15
CH25	1	S1_38997919	8.0	54.4	21.0	3.0×10^{-7}	0.17
UCU24	1	S1_39663110	10.1	44.7	25.7	8.4×10^{-12}	0.30
UCU25	1	S1_39663110	9.9	41.1	25.3	7.1×10^{-11}	0.23
CP25	1	S1_38997919	15.0	55.5	35.7	7.5×10^{-16}	0.32
BD12	4	S4_16824752	9.4	33.2	24.0	1.0×10^{-3}	0.07
BD13	4	S4_15055783	10.2	38.3	25.5	6.3×10^{-6}	0.12
BD14	4	S4_15055783	14.4	64.3	34.0	1.4×10^{-6}	0.12
BD21	1	S1_38992466	9.4	41.2	23.8	3.8×10^{-12}	0.26
BD22	1	S1_38997919	13.8	46.7	33.0	1.5×10^{-11}	0.25
BD23	1	S1_39663110	11.4	43.0	28.2	8.0×10^{-10}	0.21
BD24	1	S1_39663110	20.5	69.6	45.0	6.5×10^{-21}	0.40
BD25	1	S1_38997919	20.9	67.3	45.4	8.8×10^{-20}	0.37
FDP12	4	S4_11831218	19.3	60.6	43.0	1.1×10^{-13}	0.27
FDP13	4	S4_11831218	10.3	27.6	26.0	2.9×10^{-8}	0.19
FDP14	4	S4_11831218	5.6	19.8	15.0	1.1×10^{-3}	0.09
FDP21	4	S4_11831218	11.7	43.9	29.9	8.1×10^{-7}	0.16
FDP22	4	S4_11831218	11.4	28.1	28.6	6.0×10^{-8}	0.20
FDP23	4	S4_11831218	7.5	27.9	19.9	3.4×10^{-3}	0.08
FDP24	4	S4_11831218	8.9	28.3	23.1	1.1×10^{-5}	0.13
FDP25	4	S4_11831218	8.6	38.5	22.1	2.5×10^{-7}	0.16
RT12	4	S4_16824752	23.8	43.6	49.7	7.4×10^{-5}	0.09
RT13	4	S4_15055783	28.0	58.0	55.8	1.5×10^{-5}	0.10
RT14	4	S4_16824752	23.2	59.8	49.1	2.2×10^{-4}	0.07
RT21	4	S4_15055783	18.6	52.1	43.3	1.1×10^{-5}	0.11
RT22	4	S4_11831218	20.2	65.9	44.7	1.6×10^{-11}	0.22
RT23	4	S4_15055783	17.4	54.8	40.3	6.7×10^{-5}	0.08
RT24	4	S4_11831218	18.4	60.1	41.5	6.3×10^{-9}	0.17
RT25	4	S4_11831218	16.8	61.1	38.5	2.1×10^{-8}	0.16

[a] CH: chill hours, UCU: Utah chill units, CP: chill portions, BD: blooming date, FDP: fruit development period, RT: ripening time. [b] 'LOD' represents the statistic obtained from the interval mapping test. [c] 'K' refers to the statistic derived from the Kruskal-Wallis test. [d] 'PEV' indicates the percentage of phenotypic variation explained by the marker. [e] Parameters used for the marker–trait association (GWAS) analysis.
