# Supplementary figures and images for: Genetic Dissection of Adaptation Traits in Apricot Through GWAS and QTL Analyses
Source: Int J Mol Sci. 2026 Jul 14;27(14):6264. doi: 10.3390/ijms27146264 (PMC13410052; doi:10.3390/ijms27146264)

QTLs of phenology in 'Bergeron' (BxC)

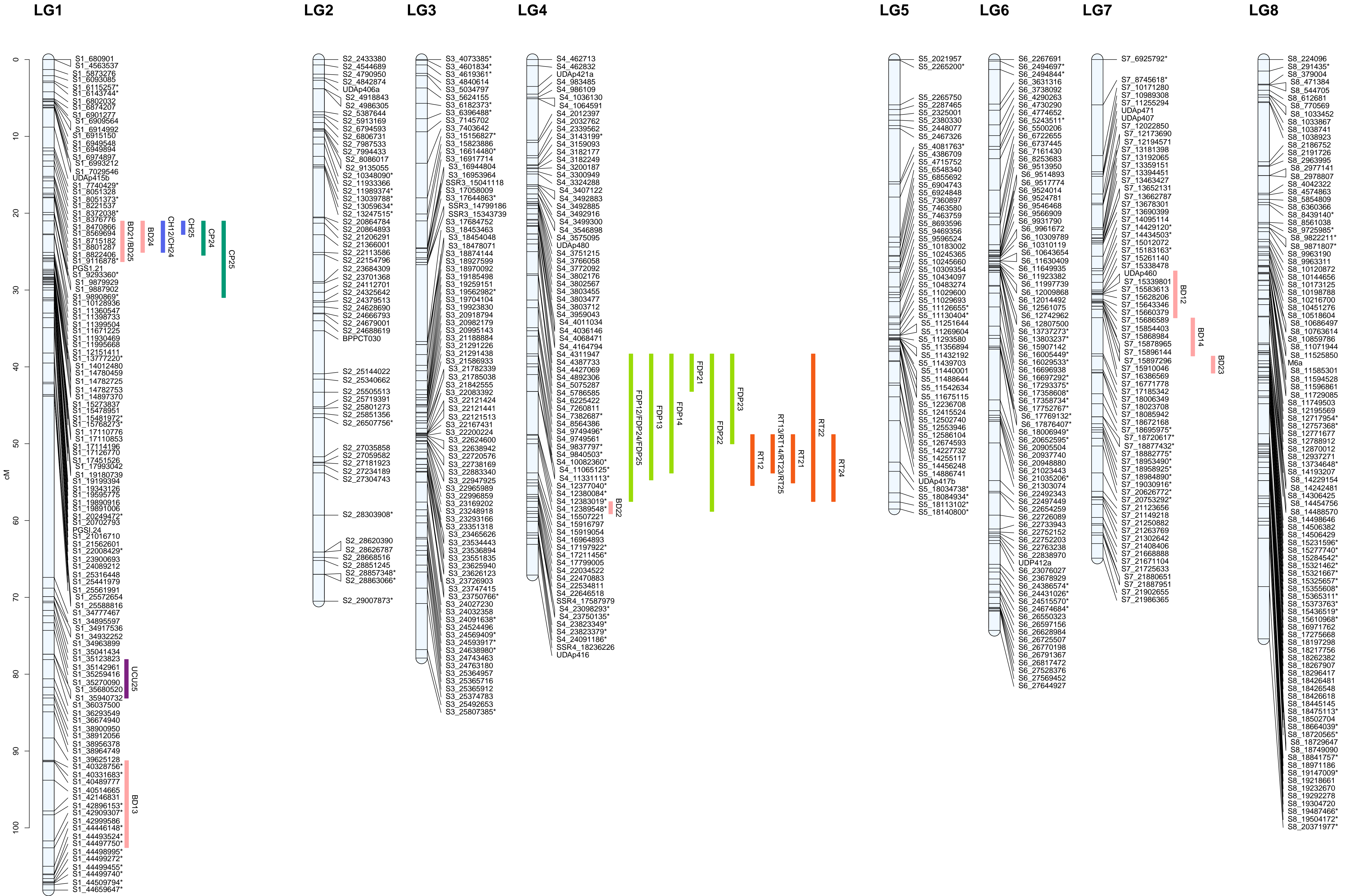

Supplement: Supplementary file 1 [file ijms-27-06264-s001.zip › ijms-4401808 R1 Figure S3.pdf]

QTLs of phenology in 'Currot' (BxC)

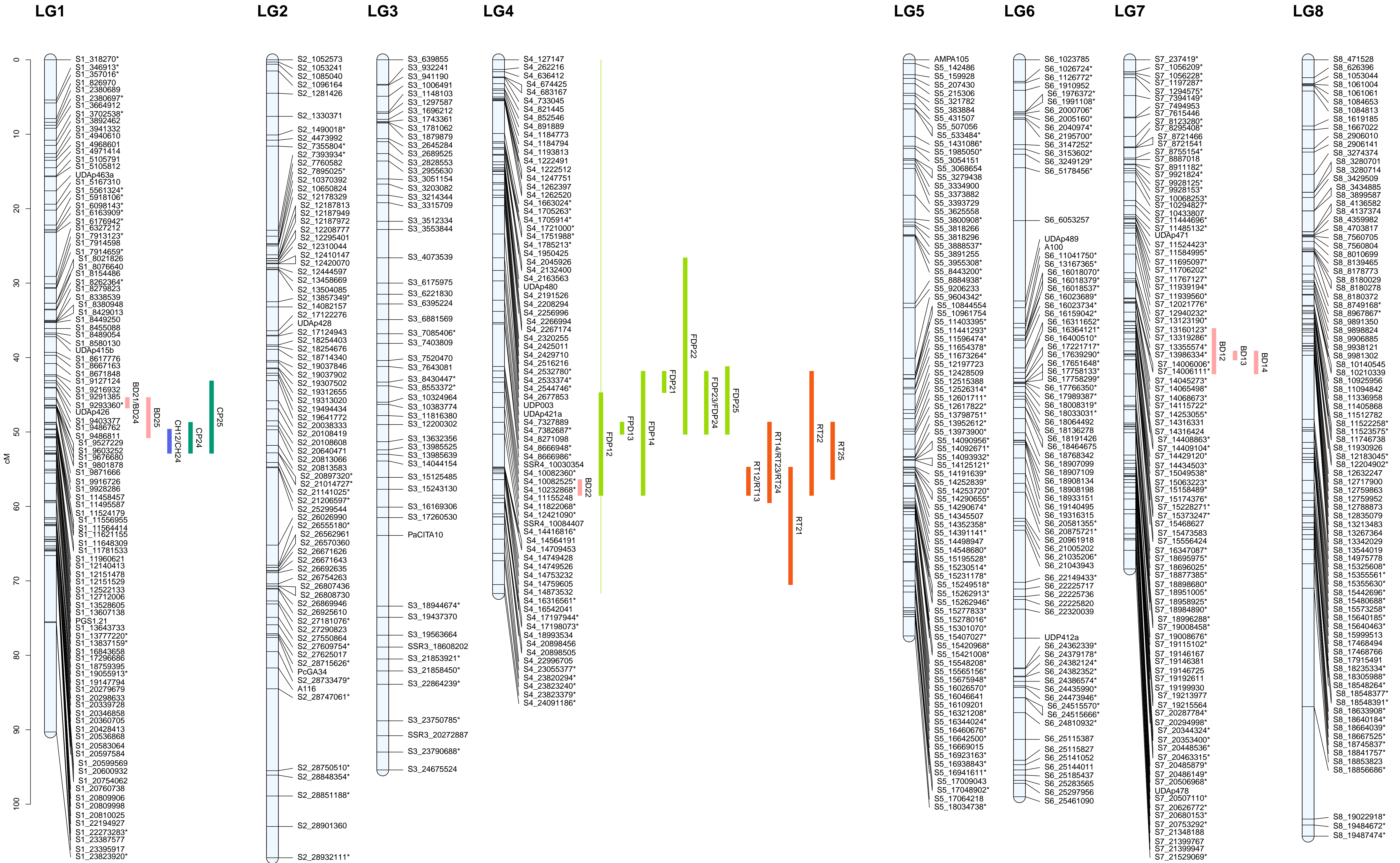

Supplement: Supplementary file 1 [file ijms-27-06264-s001.zip › ijms-4401808 R1 Figure S4.pdf]

QTLs of phenology in 'Goldrich' (GxC)

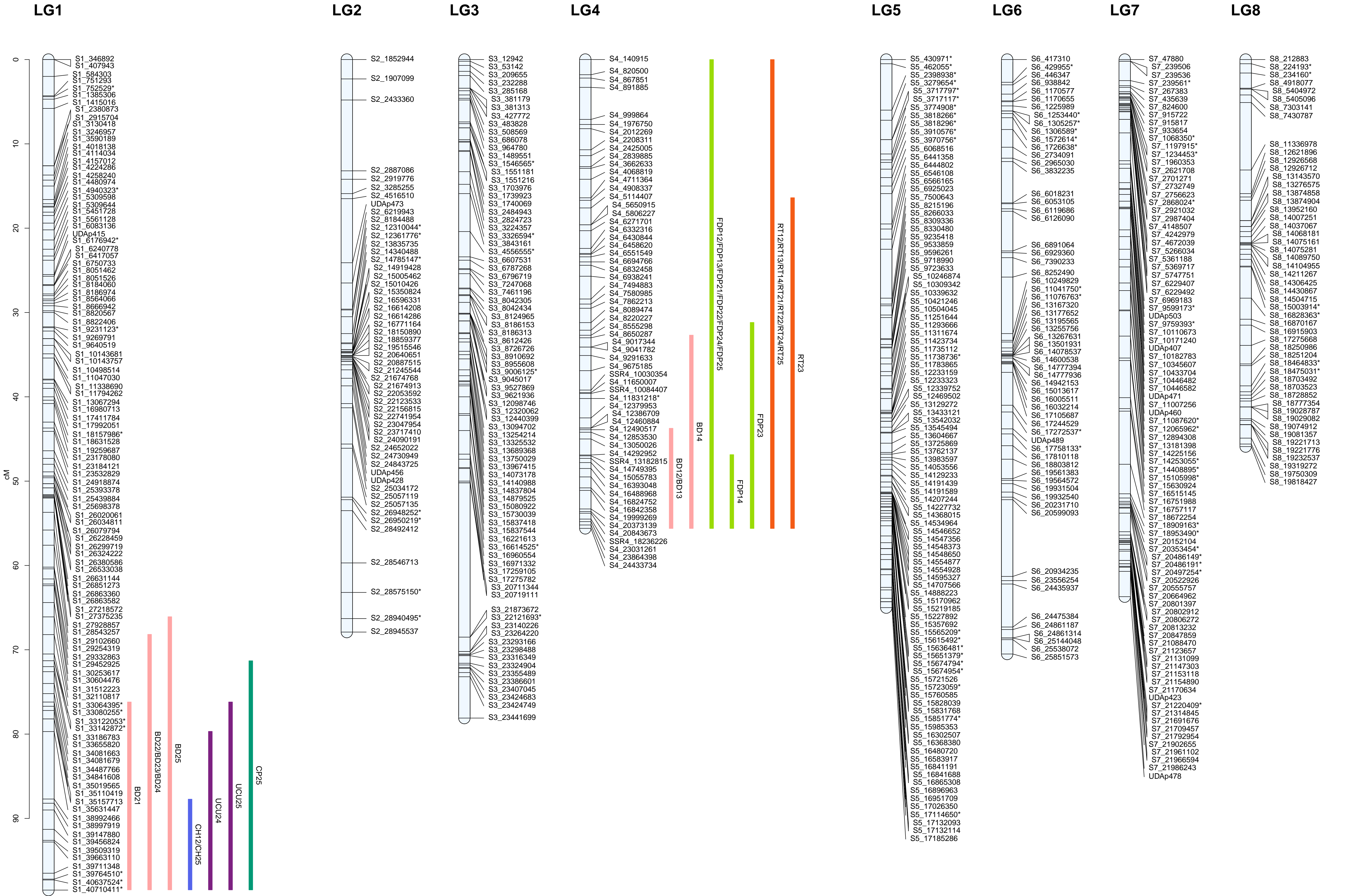

Supplement: Supplementary file 1 [file ijms-27-06264-s001.zip › ijms-4401808 R1 Figure S5.pdf]

QTLs of phenology in 'Currot' (GxC)

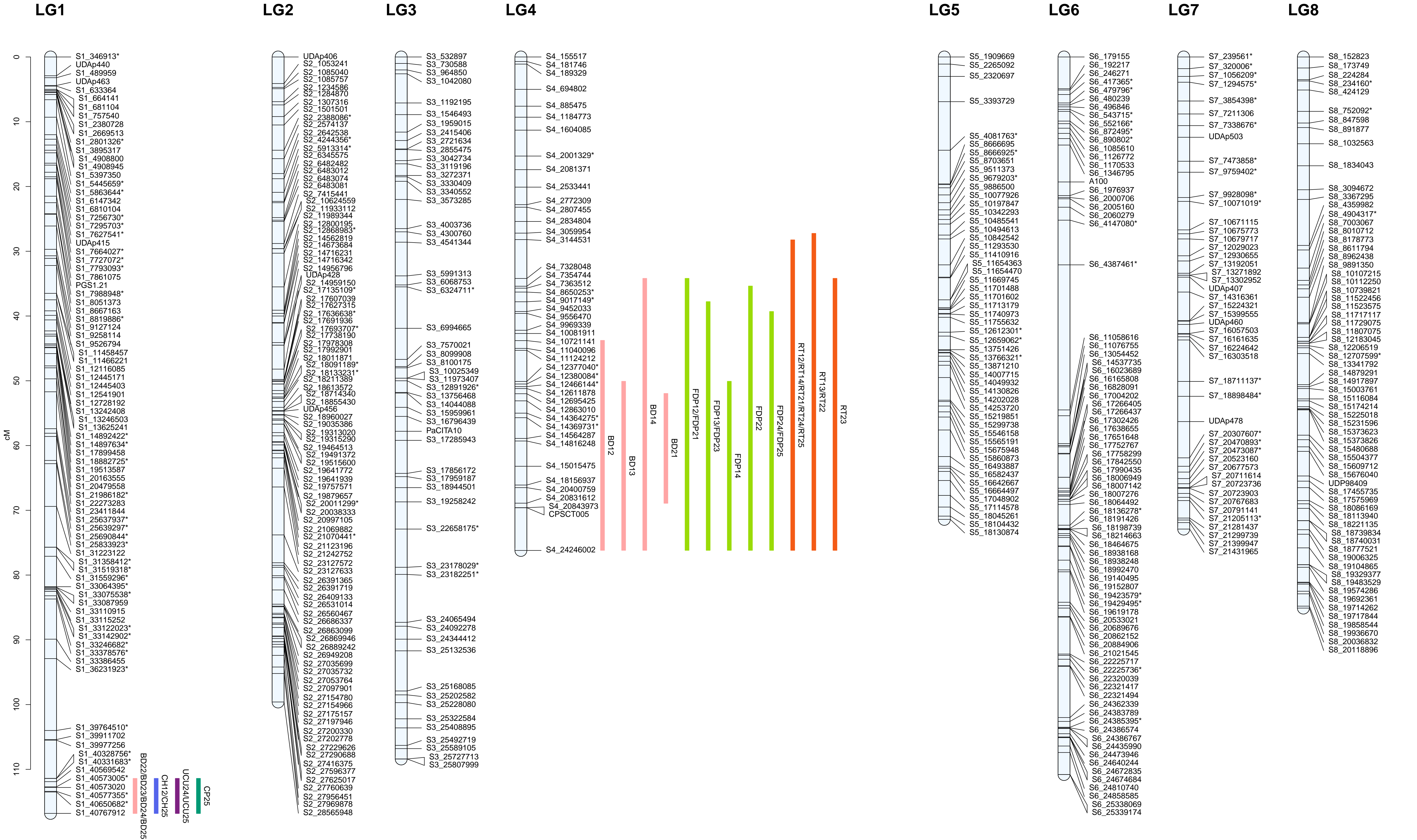

Supplement: Supplementary file 1 [file ijms-27-06264-s001.zip › ijms-4401808 R1 Figure S6.pdf]
